# Supplementary material for: The predatory bug Orius strigicollis shows a preference for egg-laying sites based on plant topography
Source: PeerJ. 2021 Jul 21;9:e11818. doi: 10.7717/peerj.11818 (PMC8308616; doi:10.7717/peerj.11818)
Supplement: Supplemental Information 1 — The diameter of the plastic screw-on lid is 13 cm, and the diameter of the central hole is nine cm. The central hole is covered with 100 mesh gauze. The filter paper is 20 cm length with 20 cm width. The total volume of mixed vermiculite (ca. 350 g) and sawdust (ca. 20 g) is 2 ± 0.5 L. [file peerj-09-11818-s001.docx]

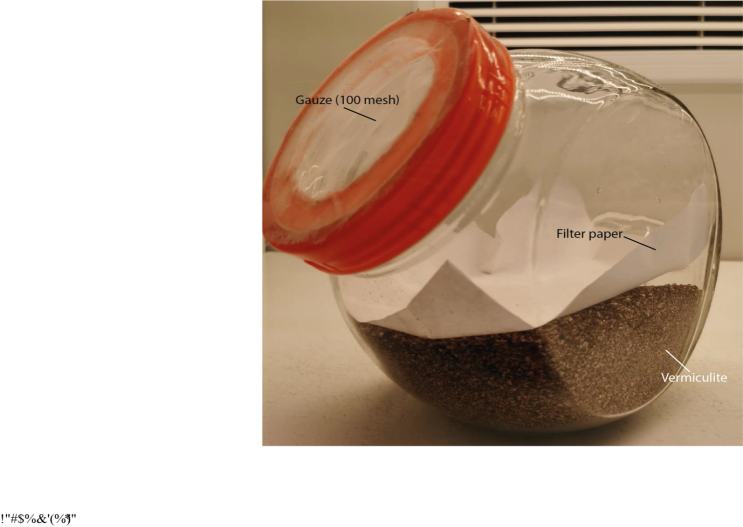


**Supplement 1: Photo of the feeding device used for all experiments (length: 16 cm, width: 22 cm, height: 23 cm, volume: 4.3 L).** The diameter of the plastic screw-on lid is 13 cm, and the diameter of the central hole is 9 cm. The central hole is covered with 100 mesh gauze. The filter paper is 20 cm length with 20 cm width. The total volume of mixed vermiculite (ca. 350 g) and sawdust (ca. 20 g) is 2 ± 0.5 L.
